# Artificial Intelligence-Enhanced Smartwatch ECG for Heart Failure-Reduced Ejection Fraction Detection by Generating 12-Lead ECG

**DOI:** 10.3390/diagnostics12030654

**Published:** 2022-03-08

**Authors:** Joon-myoung Kwon, Yong-Yeon Jo, Soo Youn Lee, Seonmi Kang, Seon-Yu Lim, Min Sung Lee, Kyung-Hee Kim

**Affiliations:** 1 Medical Research Team, Medical AI, Inc., San Francisco, CA 94103, USA; happywithhj@gmail.com (J.-m.K.); yy.jo@medicalai.com (Y.-Y.J.); seonmikang@medicalai.com (S.K.); imsun211@medicalai.com (S.-Y.L.); lylm@medicalai.com (M.S.L.); 2 Artificial Intelligence and Big Data Research Center, Sejong Medical Research Institute, Bucheon 14754, Korea; leesy@sejongh.co.kr; 3 Department of Critical Care and Emergency Medicine, Incheon Sejong Hospital, Incheon 21080, Korea; 4 Medical R&D Center, Body Friend, Co., Ltd., Seoul 06302, Korea; 5 Division of Cardiology, Cardiovascular Center, Incheon Sejong Hospital, Incheon 21080, Korea

**Keywords:** heart failure, electrocardiography, deep learning, artificial intelligence

## Abstract

Background: We developed and validated an artificial intelligence (AI)-enabled smartwatch ECG to detect heart failure-reduced ejection fraction (HFrEF). Methods: This was a cohort study involving two hospitals (A and B). We developed the AI in two steps. First, we developed an AI model (ECGT2T) to synthesize ten-lead ECG from the asynchronized 2-lead ECG (Lead I and II). ECGT2T is a deep learning model based on a generative adversarial network, which translates source ECGs to reference ECGs by learning styles of the reference ECGs. For this, we included adult patients aged ≥18 years from hospital A with at least one digitally stored 12-lead ECG. Second, we developed an AI model to detect HFrEF using a 10 s 12-lead ECG. The AI model was based on convolutional neural network. For this, we included adult patients who underwent ECG and echocardiography within 14 days. To validate the AI, we included adult patients from hospital B who underwent two-lead smartwatch ECG and echocardiography on the same day. The AI model generates a 10 s 12-lead ECG from a two-lead smartwatch ECG using ECGT2T and detects HFrEF using the generated 12-lead ECG. Results: We included 137,673 patients with 458,745 ECGs and 38,643 patients with 88,900 ECGs from hospital A for developing the ECGT2T and HFrEF detection models, respectively. The area under the receiver operating characteristic curve of AI for detecting HFrEF using smartwatch ECG was 0.934 (95% confidence interval 0.913–0.955) with 755 patients from hospital B. The sensitivity, specificity, positive predictive value, and negative predictive value of AI were 0.897, 0.860, 0.258, and 0.994, respectively. Conclusions: An AI-enabled smartwatch 2-lead ECG could detect HFrEF with reasonable performance.

## 1. Introduction

Heart failure (HF) is a significant healthcare burden worldwide, with an estimated 64.3 million people living with HF [1,2]. Despite advances in treatment, HF remains as a high risk of morbidity and mortality and is the most common diagnosis in hospitalized patients aged over 65 years, with a 5-year survival rate of only 57% [3,4,5]. In the United States, HF affects ~$30.7 billion total annual costs and projection suggests that by 2030, the total cost of HF will increase by 127%, to $69.8 billion [3,6]. 

Patients suffering with HF with reduced ejection fraction (HFrEF) become less active, leading to repeated hospitalization, resulting in a poor quality of life, including a high medical cost burden [7]. Despite its poor prognosis and high economic burden, HFrEF awareness remains relatively low due to its insidious onset, varied presentation, and syndromic nature [8]. Early diagnosis and timely intervention may prevent irreversible HFrEF progression and mortality [9]. Electrocardiography (ECG) is a low-cost test frequently performed for a variety of purposes, especially basic examination and screening for cardiovascular disease [10]. We developed an artificial intelligence (AI)-enabled ECG algorithm, which can increase the diagnosis of HFrEF [11,12]. However, it is also inconvenient to visit the hospital for a 12-lead ECG.

Smartwatches have high processing power and sophisticated sensors that can provide new health data, including ECG. In this study, we developed and validated an artificial intelligence-enabled smartwatch ECG for HFrEF detection. As the smartwatch could obtain a single lead ECG, we also developed a deep learning-based model (DLM) for generating a 12-lead ECG from a smartwatch ECG (Lead I and II) to enhance AI performance and detect HFrEF using the generated 12-lead ECG. Moreover, we conducted internal and external validation for the developed AI in this multicenter study. To the best of our knowledge, this is the first study to generate a 12-lead ECG from a smartwatch ECG and detect heart failure. Based on this deep learning model, heart failure with reduced ejection fraction could be detected in daily living by using asynchronous 2-lead ECGs from lifestyle ECG devices, such as smart watches.

## 2. Methods

### 2.1. Data Source and Study Population

**Figure 1:**
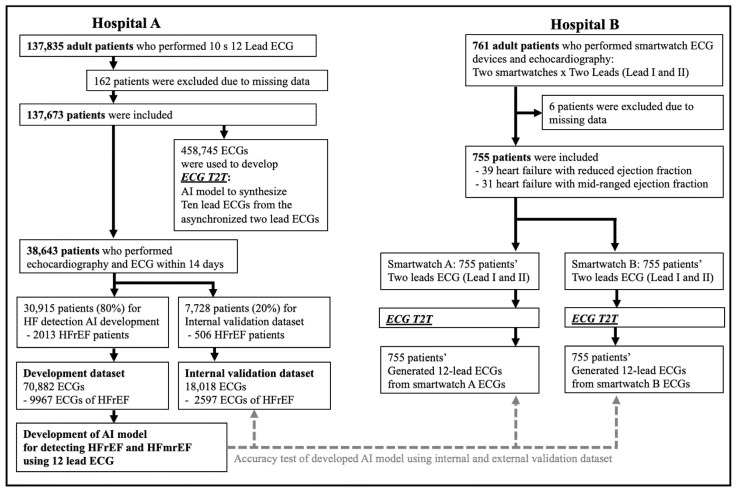
Study flowchart. Legend: AI denotes artificial intelligence, ECG electrocardiography, ECGT2T ECG synthesis from two-lead to ten-lead, HF heart failure, HFmrEF heart failure with mildly reduced ejection fraction, and HFrEF heart failure with reduced ejection fraction.

We conducted a multicenter retrospective cohort study to develop and validate the AI in two hospitals, as shown in Figure 1. Hospital A is a cardiovascular teaching hospital, and hospital B is a community general hospital. Data from hospital A were used for developing the AI and for internal performance tests, and data from hospital B for external performance tests with smartwatch ECG. First, we included all adult patients aged 18 years and older who underwent at least one 10 s 12-lead ECG at hospital A during the study period (1 November 2015–31 May 2021). The 10 s 12-lead ECG was acquired in the supine position and digitally stored at a 500 Hz sampling rate. The data were used to develop the AI models, with ECGT2T (ECG synthesis from two-lead to ten-lead) being used to generate the 12-lead ECG from two non-synchronized lead (Lead I and II) ECG. Second, we included adult patients aged 18 years and older who underwent both 10 s 12-lead ECG and echocardiography within 14 days at hospital A during the study period (1 November 2015–30 June 2021). The data were split into development data (80%) to develop the AI model for detecting HFrEF using 12-lead ECG and internal performance test data (20%). Third, for the external test dataset (smartwatch ECG), we included adult patients aged 18 years and older who underwent smartwatch ECG and echocardiography in the study period (1 June 2021–30 July 2021). We used two types of smartwatches—Galaxy Watch Active (Smart watch A) and Apple Watch 6 (Smart watch B). Two lead (Leads I and II) were obtained from each patient using each smartwatch. The method to obtain two 2-lead ECG using a smartwatch was described in a previous study [13]. The study population with missing clinical information, including ECG, echocardiographic results, or demographic data, was excluded. The Bucheon and Incheon Sejong Hospital Institutional Review Board approved this study protocol and waived the need for informed consent due to minimal harm and impracticality. This study complied with the Declaration of Helsinki.

### 2.2. Outcomes and Predictive Variables

The primary outcome of this study was the performance of the AI in detecting patients with HFrEF using a standard 10 s 12-lead ECG or smartwatch ECG. HFrEF was defined as an ejection fraction of 40% or less on transthoracic comprehensive echocardiography, which was recorded in the electronic health record database at the time of acquisition, symptom, and signs from medical records [14]. The EF was determined using a biplane approach with the Simpson and 2D methods. If the estimated EF was in a range, we used the middle value as a single EF value. If more than two echocardiographies were obtained within 14 days from the ECG, we used echocardiography that was closest to ECG as index echocardiography. The secondary outcome was the performance of the AI in detecting patients with HF with mildly reduced EF (HFmrEF) to reduced EF (<50%) on echocardiography [14]. Predictive variables were ECG, age, sex, weight, and height.

### 2.3. Data Preprocessing

We preprocessed the ECGs for sampling, normalization, and augmentations. We constructed an ECG with 8 s by cropping of 1 s on each side and normalized (z-score) based on the mean and standard deviation. In terms of augmentations, the addition of linear and nonlinear noise causing baseline changes was performed. We also normalized the values of age, weight, and height. We changed the value of sex to one-hot encoding.

### 2.4. Development for a Platform Detecting HFrEF 

**Figure 2:**
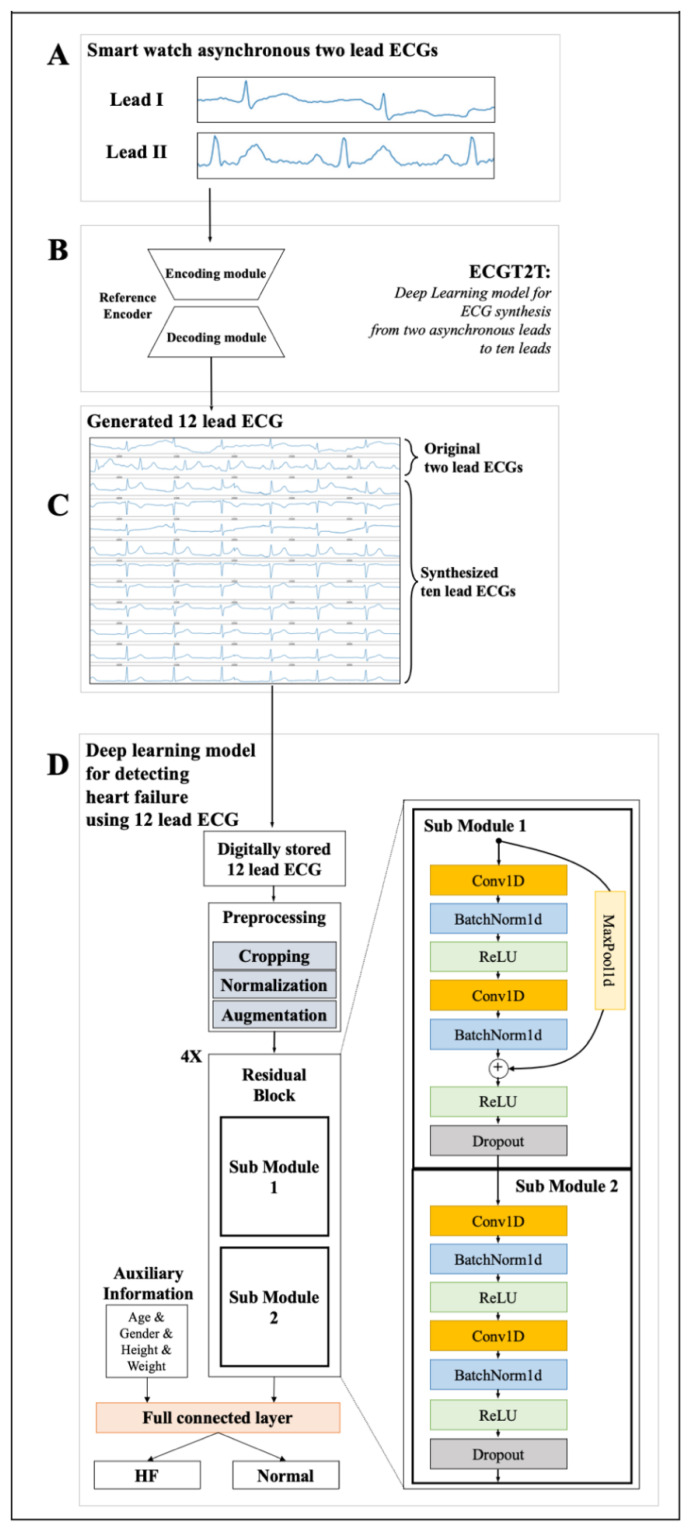
Architecture of deep learning model for detecting heart failure. Legend: ECG denotes electrocardiography, ECGT2T ECG synthesis from two-lead to ten-lead, and HF heart failure. (**A**) Asynchronous two lead ECGs from smart watch. (**B**) ECGT2T for synthesizing ten lead ECG from two lead ECG. (**C**) Generated twelve lead ECG which input to final AI model. (**D**) Deep learning model for detecting heart failure with reduced ejection fraction using generated twelve lead ECG.

Our AI consists of two phases. First phase generates a standard 12-lead ECG. We developed an ECGT2T for generating a 12-lead ECG from an asynchronous 2-lead ECG, as shown in Figure 2. ECGT2T is a deep learning model based on a generative adversarial network that synthesizes a 10-lead ECG (III, aVR, aVL, aVF, and V1–6) from an asynchronous 2-lead ECG (leads I and II). It translates source ECGs to reference ECGs by learning the styles of reference ECGs; it first generates a single latent code representing the cardiac condition from two given leads and then reconstructs the other 10 leads by referring to the single latent code based on the single given lead. For ECGT2T development, development data from hospital A were used. We have described the detailed development process of ECGT2T in our previous article [15]

Second phase detects a HFrEF. We developed another deep learning model based on four residual blocks for detecting HFrEF using a 12-lead ECG. This model was trained using the development dataset from hospital A. Figure 2 show the architecture of the HFrEF detection model. Each residual block contains two submodules, each of which has a one-dimensional convolutional neural network, batch normalization, rectified linear unit activation, and a dropout layer. The difference between both sub modules is the skip connection. The first sub module has only the skip connection. The latent variables passing through all residual blocks are concatenated to auxiliary information including age, gender, height, and weight. Thereafter, the final output was obtained through two fully connected layers. We used the Adam optimizer, which is a popular algorithm in the field of deep learning because it achieves good results fast. Additionally, we found that its performance was better than that of SGD, RMSprop, or Adadelta in the pilot study. For parameter settings, we set the learning rate to 1 × 10^−4^, weight decay to 1 × 10^−5^, epsilon to 1× 10^−8^, beta1 to 0.9, and beta2 to 0.999, respectively. We confirmed the values of parameters by grid search methods. We selected the search space of grid search by using random sampling search of pilot study. We train the models on a high-performance computer composed of 20 DGX servers with 160 NVIDIA A100 graphics processing units. All models were developed using PyTorch and Python. 

### 2.5. Statistical Analysis

We used the area under the receiver operating characteristic curve (AUC) to confirm the performance based on the continuous prediction score and presence of HFrEF. The 95% confidence intervals (CIs) of AUC were confirmed by the Sun and Su optimization of the DeLong method. We confirmed the sensitivity, specificity, positive predictive value (PPV), and negative predictive value (NPV) using a cut-off point. The cut-off point was defined using Youden’s J statistics [16]. We used two-sided 95% CIs to describe the variability of the study population and estimates. We used exact CIs based on Clopper–Pearson to be conservative for accuracy, sensitivity, specificity, PPV, and NPV. We analyzed the statistical results using R version 3.4.3.

### 2.6. Role of the Funding Sources

This study was supported by a National Research Foundation of Korea grant funded by the Korean government (No. 2020R1F1A1073791). None of the listed entities played any role in the design of the study; data collection, model development, result interpretation, writing article, or decision to submit this paper. All authors had full access to the data and the final decision of submission. 

## 3. Results

We identified 137,835 patients from hospital A and after applying exclusion criteria, including 137,673 patients with 458,745 ECG for development dataset for developing ECGT2T. Among 137,673 patients from hospital A, 38,643 adult patients who underwent both 10 s 12-lead ECG and echocardiography within 14 days at hospital A were included to develop model to detect HFrEF using a 10 s 12-lead ECG. For external testing to confirm the performance for detecting HFrEF using smartwatch ECG, 761 patients from hospital B were identified and 6 patients were excluded due to missing values of ECG and echocardiography. Finally, 755 patients with 1510 ECG dataset (2-lead ECG from smart watch A and B) were included in the external performance test. As shown in Table 1, the HFrEF patients were older than the non-HFrEF patients. And HFrEF patients had more prolonged QRS interval and atrial fibrillation or flutter than the non-HFrEF patients.

**Figure 3:**
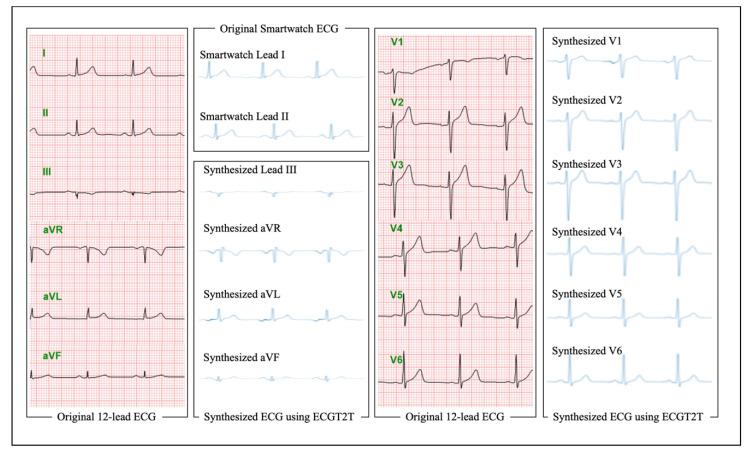
12-lead ECG generation using smartwatch ECG based on ECGT2T. Legend: ECG denotes electrocardiography and ECGT2T ECG synthesis from two-lead to ten-lead.

**Figure 4:**
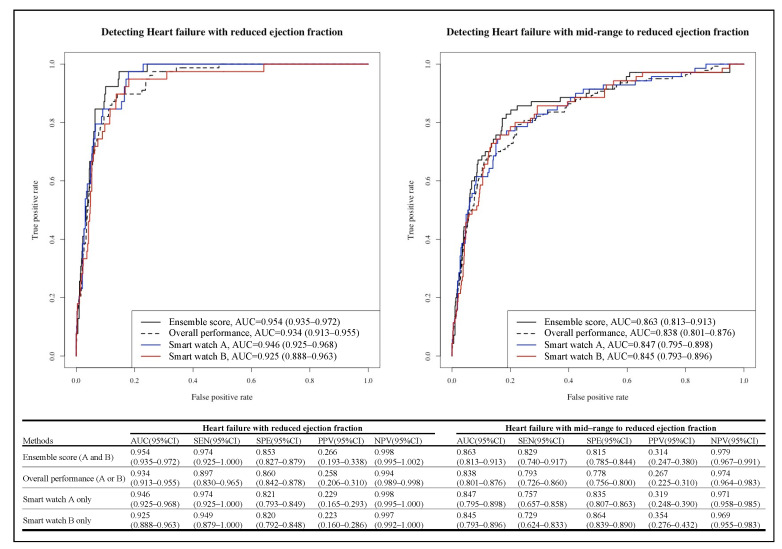
Performance of artificial intelligence for detecting heart failure using smart watch ECG. Legend: AUC denotes area under the receiver operating characteristic curve, CI confidence interval, ECG electrocardiography, NPV negative predictive value, PPV positive predictive value, SEN sensitivity, and SPE specificity.

During internal validation of ECGs (hospital A), the AUC of the AI model to detect HFrEF and HFmrEF to HFrEF using a 10 s 12-lead ECG were 0.934 (0.929–0.938) and 0.909 (0.904–0.914), respectively. As shown in Figure 3, the ECGT2T generated a 10 s 12-lead ECG using a smartwatch 2-lead ECG (Lead I and II). We input the generated 10 s 12-lead ECG to develop an AI model that detects HFrEF using a 12-lead ECG. During external validation (hospital B), the AUC of AI for detecting HFrEF using ECG from smartwatch A and B were 0.946 (0.925–0.968) and 0.925 (0.888–0.963), respectively. The overall performance of 1510 ECG datasets (755 smartwatch A and 755 smartwatch B) was 0.934 (0.913–0.955), as shown in Figure 4. The AUC of ensemble score, defined as the average of the prediction scores of smartwatch A and B, was 0.954 (0.935–0.972). The sensitivity, specificity, PPV, and NPV of the AI model in smartwatch A were 0.974 (0.925–1.000), 0.821 (0.793–0.849), 0.229 (0.165–0.293), and 0.998 (0.995–1.000), respectively. The sensitivity, specificity, PPV, and NPV of the AI model in smartwatch B were 0.949 (0.879–1.000), 0.820 (0.792–0.848), 0.223 (0.160–0.286), and 0.997 (0.992–1.000), respectively. During external validation (hospital B) of secondary output, the AUC of AI for detecting HFmrEF to HFrEF using ECG from smartwatch A and B were 0.847 (0.795–0.898) and 0.845 (0.793–0.896), respectively.

## 4. Discussion

In this study, we proposed AI-enabled smartwatch ECG to detect HFrEF and it showed reasonable performance as a screening tool. These results outperformed other screening tools, such as B-type natriuretic peptide for HFrEF (AUC 0.871) [17]. This study showed the feasibility of using a smartwatch to diagnose diverse diseases other than arrhythmia. As this smartwatch is already used in our daily lives, we could monitor and detect HFrEF patients using our proposed AI model.

The ECG of each lead is a signal for measuring the electrical flow of the heart in each lead vector. The electrical vector of the heart can be estimated by synthesizing the ECG data of the two leads. A 12-lead ECG can be generated by reconstructing the ECG corresponding to the vector of each lead, determined based on the estimated electrical flow of the heart. ECGT2T is a deep learning model based on this concept and generates an ECG using a generative adversarial network.

Twelve-lead ECG was required for evaluating cardiovascular disease status using diverse vector information of the heart. However, in daily living, 12-lead ECG is not always practical or feasible because it is difficult to place the chest and limb lead at the exact location. In this study, we generated 12-lead ECG and detected heart failure with reduced ejection fraction using only leads I and II, which could be captured by a smart watch in use in daily living. Therefore, this study is a milestone in using ECG to detect cardiovascular disease in daily living. Although previous studies have detected diseases using ECG, studies using 12-lead ECG and other devices have been conducted only in hospital settings. Here, we developed a deep learning methodology that generates ECGs from lifestyle devices and smartwatches. Therefore, our methodology can be used in daily living. The major contribution of this study is to provide a methodology for detecting disease in daily living based on deep learning model using a single lead life style device, such as a smart watch.

The increasing prevalence of HF making it among the most costly diseases to Medicare [18]. More than 30% HF patients are seen in the clinic setting, and more than 40% of those recently admitted with decompensation will require a second hospitalization within a year [19]. Early detection of HFrEF offers the opportunity to test and develop an effective lifestyle and life-saving medical therapy [9]. The evolution and adoption of digital health technology and mobile health devices may address this issue. Our day-to-day lives are impacted by technological innovations, and the recent trend of commercial smart wearable devices aims in improving our health [20]. Smart wearables are connected electronic devices designed for everyday use that can be worn on the body as an accessory or integrated into clothing. Smartwatches and wristbands have high processing power and sophisticated sensors that can provide new health information [20]. Wearable health devices are an aspect of medical health that may improve the delivery of HF care by allowing medical data collection outside of a clinician’s office or hospital. Wearable devices are externally applied and capture functional or physiological data to monitor and improve patients’ health. It could be a cost-effective method to detect HF before it becomes fatal. Personalized patient care has become remote and decentralized owing to the COVID-19 pandemic [21]. The cardiovascular community must utilize the commercially available wearable technologies as well as the wide range of clinical applications that they can serve. This technology integration into the clinical workplace, however, is still in its early stages.

Per previous medical knowledge, ECGs could be used to detect small coverage diseases, such as arrhythmia and ST-segment elevation myocardial infarction. We could not develop diagnostic criteria and tools using non-linear correlations between diverse diseases and subtle changes based on conventional statistical methods, such as logistic regression [22]. Recently, AI has been adopted to diagnose many diseases and conditions and to predict the development of disease [23]. Most AI for detecting diseases using ECG is based on deep learning. The most important strength of deep learning is the automatic feature extraction [24]. Specifically, deep learning automatically extracts the features of ECG to detect disease, without any human engineering resource needed to define the features for using the model. This has reduced the time and cost of AI development. The importance of automatic feature extraction is that we can extract features and develop a model without human prejudice. AI based on deep learning is based only on information from data, not medical knowledge. This aspect showed the possibility of enhancing the model to detect diverse diseases and show new medical findings over previous medical knowledge.

An important pitfall of deep learning is overfitting [23]. Especially, deep learning can be subjugated to the environment in which development data are obtained. Therefore, we should validate the developed AI to other hospitals and environments. The important point of this study was external validation. We confirmed the performance of the AI model using data from other hospitals and other devices in this study. In other word, we developed AI using 12-lead ECG data from hospital A and external validated AI using smartwatch ECG from hospital B.

The advantage of AI is that it saves healthcare costs. Using this AI, HFrEF could be screened by a wearable watch without a physician, and it could refer patients at risk to cardiologists for confirmative diagnostic tests. Therefore, this procedure is advantageous for low-income countries to save patients from irreversible disease progression and death. This AI could be used in wearable watches in daily living and HFrEF could be detected and monitored in the early stages without complications. This predictive care solution is essential to reduce healthcare costs.

This study had several limitations. First, we validated the AI-enabled smartwatch ECG to detect HFrEF in a hospital setting. As there is a possibility of decreasing performance in daily living at home, we needed to validate this AI in a home setting [25]. Second, this study was conducted in South Korea, and it is necessary to validate the AI in other countries. We will verify the performance and significance of the proposed model through a prospective remote home care setting in a multinational study.

## Figures and Tables

**Table 1:** Baseline characteristics table.

	Hospital A (38,643 Patients) Development and Interval Validation Data				Hospital B (755 Patients) External Validation Smart Watch Data				
Characteristics	HFrEF	HFmrEF	Normal	*p* †	HFrEF	HFmrEF	Normal	*p* †	*p* ‡
Total Patients	2519 (6.5)	1755 (4.5)	34,369 (88.9)		39 (5.2)	31 (4.1)	685 (90.7)		0.241
Age (year)	64.82 (13.52)	64.98 (13.47)	58.60 (15.45)	<0.001	60.69 (13.84)	58.97 (14.46)	55.61 (15.16)	0.067	<0.001
Male	1665 (65.7)	1083 (61.3)	16,341 (47.6)	<0.001	29 (74.4)	20 (64.5)	325 (47.4)	0.001	0.969
Weight (Kg)	64.68 (13.90)	65.25 (13.34)	64.87 (12.60)	0.341	69.29 (14.02)	66.80 (12.68)	66.03 (14.07)	0.358	0.004
Height (cm)	162.68 (9.56)	162.37 (10.01)	162.20 (9.58)	0.044	168.41 (10.55)	164.48 (9.36)	163.38 (9.39)	0.005	<0.001
Body surface area (m^2^)	1.70 (0.22)	1.71 (0.21)	1.70 (0.20)	0.505	1.79 (0.22)	1.74 (0.20)	1.72 (0.22)	0.145	0.001
Heart rate (bpm)	84.37 (24.54)	78.26 (20.53)	73.14 (15.82)	<0.001	78.31 (19.78)	70.03 (14.55)	69.95 (12.56)	0.001	<0.001
PR interval (ms)	175.83 (36.69)	176.83 (37.36)	167.99 (29.01)	<0.001	122.00 (60.53)	156.74 (144.40)	149.46 (97.07)	0.225	<0.001
QRS duration (ms)	111.81 (27.97)	104.85 (23.55)	95.47 (15.88)	<0.001	155.74 (63.39)	139.42 (31.17)	138.95 (31.03)	0.010	<0.001
QT interval (ms)	407.78 (57.74)	408.08 (51.59)	398.94 (40.13)	<0.001	421.90 (99.88)	417.63 (45.29)	425.56 (50.21)	0.681	<0.001
Atrial fibrillation of flutter	296 (11.7)	170 (9.6)	1172 (3.4)	<0.001	3 (7.7)	1 (3.2)	14 (2.0)	0.076	0.015
P wave axis	45.58 (39.72)	44.48 (35.84)	45.32 (28.89)	0.585	NA	NA	NA	NA	
R wave axis	27.71 (65.00)	31.15 (53.61)	39.80 (42.07)	<0.001	NA	NA	NA	NA	
T wave axis	83.07 (85.26)	58.82 (72.34)	42.57 (44.37)	<0.001	NA	NA	NA	NA	
Ejection fraction (%)	32.03 (9.44)	46.08 (5.98)	60.64 (6.33)	<0.001	31.23 (7.21)	45.97 (2.50)	64.63 (5.19)	<0.001	<0.001
Left atrial dimension (mm)	45.66 (8.97)	44.05 (9.48)	38.98 (7.84)	<0.001	43.76 (7.48)	42.48 (9.31)	36.48 (6.90)	<0.001	<0.001
E	67.69 (27.32)	63.05 (25.71)	63.50 (19.49)	<0.001	72.00 (22.11)	68.65 (27.26)	66.55 (19.21)	0.37	<0.001
A	68.40 (23.50)	71.03 (21.03)	70.06 (20.23)	0.002	71.28 (22.05)	74.00 (25.71)	66.74 (20.64)	0.251	<0.001
E′	5.04 (1.91)	5.72 (2.09)	7.10 (2.67)	<0.001	5.80 (3.81)	6.06 (2.54)	7.64 (4.62)	0.044	<0.001
E/E′	14.90 (7.84)	12.04 (6.27)	9.88 (4.58)	<0.001	15.37 (6.90)	13.13 (7.88)	9.85 (4.22)	<0.001	0.534

† The alternative hypothesis for this *p* value was that there was a difference between the heart failure with reduced ejection fraction, heart failure with mildly reduced ejection fraction, and non-heart failure. ‡ The alternative hypothesis for this *p* value was that there is a difference between hospital A (derivation and internal validation data group) and hospital B (external validation group) for each variable.

## Data Availability

The data underlying this article will be shared on reasonable request to the corresponding author.
